# Transient Thyroiditis Followed by Exacerbation of Hypothyroidism After Immune Checkpoint Inhibitor Therapy (Nivolumab and Ipilimumab) in a Patient With Pre-existing Autoimmune Hypothyroidism: A Case Report

**DOI:** 10.7759/cureus.39439

**Published:** 2023-05-24

**Authors:** Guy Hayakawa, Maya M Leibowitz, Sudipta Datta, Samson O Oyibo

**Affiliations:** 1 General Medicine, Peterborough City Hospital, Peterborough, GBR; 2 Oncology, Peterborough City Hospital, Peterborough, GBR; 3 Diabetes and Endocrinology, Peterborough City Hospital, Peterborough, GBR

**Keywords:** combination therapy, levothyroxine, autoimmune hypothyroidism, transient thyroiditis, immune checkpoint inhibitors, ipilimumab, nivolumab

## Abstract

Treatment with immune checkpoint inhibitors has improved the prognosis of solid tumors. However, immune-related adverse events (IRAEs), including exacerbation of pre-existing autoimmune disease, are common and have become more frequent with combination therapy. The literature is scanty regarding reports of the use of combination immune checkpoint therapy in patients with pre-existing autoimmune hypothyroidism. We report a case of a man with a history of hypothyroidism, who developed transient thyroiditis, characterized by a thyrotoxic phase followed by a severe hypothyroid phase soon after receiving combination therapy (nivolumab and ipilimumab) for the treatment of a malignant pleural mesothelioma. He had been on a stable low dose of levothyroxine for 12 years prior to this episode. His levothyroxine requirement markedly increased soon after the episode of immune checkpoint inhibitor-induced thyroiditis. Immune checkpoint inhibitors can cause destructive thyroiditis followed by exacerbation of hypothyroidism in patients with pre-existing autoimmune hypothyroidism, such that patients end up on a higher dose of levothyroxine. This case will add to the growing literature regarding thyroid IRAEs associated with the use of immune checkpoint inhibitors in patients with pre-existing autoimmune thyroid disease.

## Introduction

The blockade of immune checkpoints with immune checkpoint inhibitor drugs has improved the prognosis of previously untreatable, unresectable solid tumors. Immune checkpoints are receptor proteins on the surface of immune cells that control immune reactions, such that unwanted reactions and autoimmunity do not occur. These receptor proteins include the programmed cell death 1 (PD-1) protein, the programmed cell death-ligand 1 (PD-L1) protein, and the cytotoxic T lymphocyte-associated protein 4 (CTLA-4). Cancer cells prevent an anti-cancer immune response by reacting with these immune checkpoint proteins [1-3].

These immune checkpoint inhibitors are monoclonal antibodies used to block immune checkpoints, such that cancer cells cannot react with these points and thus inhibit an effective anti-cancer response [1-3]. Several of these immune checkpoint inhibitors are being used with growing success for the treatment of solid tumors. Nivolumab and pembrolizumab are anti-PD-1 agents; atezolizumab, durvalumab, and avelumab are anti-PD-L1 agents; and ipilimumab and tremelimumab are anti-CTLA-4 agents [4].

Immune-related adverse events (IRAEs) are a common problem associated with the use of immune checkpoint inhibitors. While rendering the immune system able to attack the cancer cells, they also remove the pre-existing checks on unwanted reactions and autoimmunity [5]. The endocrine system is commonly affected, with thyroid gland dysfunction being the commonest type of endocrine IRAEs affecting 5%-20% of individuals on immune checkpoint inhibitor therapy, with the highest incidence occurring in those on combination therapy [6,7]. Affected individuals usually develop severe destructive thyroiditis followed by hypothyroidism, but there are reports of individuals developing new Graves’ hyperthyroidism [6,7].

Patients with pre-existing autoimmune disease are more commonly affected by IRAEs when compared to those without. This also includes a flare-up of the pre-existing autoimmune disease [8]. Such immune checkpoint inhibitor therapy-induced flare-ups are usually mild and require only conservative therapy [9].

The literature is scanty regarding reports of the effect of combination immune checkpoint inhibitor therapy in patients with pre-existing autoimmune hypothyroidism. We report a case of a man with pre-existing autoimmune hypothyroidism, who developed transient thyroiditis characterized by a moderate-to-severe thyrotoxic phase, followed by a severe exacerbation of the pre-existing hypothyroidism after initiation of two immune checkpoint inhibitors (nivolumab and ipilimumab) for the treatment of a malignant pleural mesothelioma. He had been stable on low-dose thyroid replacement therapy for 12 years prior to starting the immune checkpoint therapy and ended up on a higher dose thereafter.

## Case presentation

Medical history and demographics

A 65-year-old man with a history of hypothyroidism and also receiving treatment for a malignant pleural mesothelioma was referred to the endocrinology department because of thyrotoxicosis. This was discovered during a routine blood test (the patient did not have any new symptoms).

He had been diagnosed with a right-sided malignant pleural mesothelioma for which he had recently received two intravenous doses of nivolumab three weeks apart and a single intravenous dose of ipilimumab. Other medical history included type 2 diabetes, ischemic heart disease, autoimmune hypothyroidism, and a right internal carotid artery occlusion. His usual medication list included levothyroxine, aspirin, atorvastatin, lisinopril, metformin, metoprolol, and omeprazole. His thyroid replacement therapy had been stable for 12 years on 50 mcg daily of levothyroxine (0.6 mcg/kg body weight). He was a chronic smoker and drank little alcohol. There were no significant findings on clinical examination, and his weight was 82 kg.

Investigations

Initial blood tests revealed thyrotoxicosis two weeks after the second dose of nivolumab. The thyroid-stimulating hormone (TSH) level was suppressed, while the free triiodothyronine (Free T3) and free thyroxine (Free T4) levels were elevated, consistent with the thyrotoxic phase of immune therapy-induced thyroiditis. His renal function, kidney function, and full blood count were normal (Table 1). His anti-thyroid peroxidase antibody levels were elevated, albeit less than the levels at the time of original diagnosis of his Hashimoto’s disease. His thyroid receptor antibody levels were normal (Table 1). A subsequent ultrasound scan demonstrated an atrophic thyroid gland with heterogeneous echogenicity and no increased vascularity.

**Table 1:** Initial blood test results Routine blood tests on follow-up visit revealing thyrotoxicosis

Blood test	Result	Reference range
Sodium (mmol/L)	135	132-145
Potassium (mmol/L)	5.0	3.4-5.1
Chloride (mmol/L)	107	97-110
Creatinine (μmol/L)	63	45-84
Cortisol (nmol/L)	329	270-700
Thyroid-stimulating hormone (mU/L)	0.02	0.3-4.2
Free thyroxine (pmol/L)	42.3	12.0-22.0
Free triiodothyronine (pmol/L)	10.3	3.1-6.8
Anti-thyroid peroxidase antibodies (IU/mL)	311	< 34
Thyroid receptor antibodies (IU/L)	< 1.5	< 2.9
Total protein (g/L)	67	60-80
Albumin (g/L)	40	35-50
Globulin (g/L)	27	20-35
Alanine transferase (U/L)	20	10-60
Alkaline phosphatase (U/L)	90	30-130
Hemoglobin (g/L)	136	115-165
White cell count (10^9^/L)	10.3	4.0-11.0
Platelet count (10^9^/L)	399	150-400

Treatment

The levothyroxine dose was initially reduced to 25 mcg daily in response to the thyrotoxic test result, and the TSH level normalized to 1.8 mU/L four weeks later (free T4 was not provided). However, a repeat blood test performed four weeks later demonstrated severe hypothyroidism (TSH level of 82.2 mU/L, free T4 level of 5.2 pmol/L). His levothyroxine dose was increased to 75 mcg daily, with a further increase to 125 mcg daily (1.5 mcg/kg body weight) over the next two months.

Outcome and follow-up

The patient remained adequately replaced on 125 mcg daily of levothyroxine and continued the immune checkpoint inhibitor therapy (nivolumab and ipilimumab). He remains well while receiving regular follow-up under both the oncologist and the endocrinologist.

## Discussion

We have described a case of a patient who had pre-existing autoimmune hypothyroidism (Hashimoto's disease) maintained on low-dose levothyroxine (0.6 mcg/kg body weight) for 12 years. However, after one dose of ipilimumab and two doses of nivolumab for a malignant pleural mesothelioma, he developed transient and destructive thyroiditis, which was characterized by a moderate-to-severe thyrotoxic phase followed by a severe hypothyroid phase, and ended up requiring a higher dose of levothyroxine (1.5 mcg/kg body weight). The patient did not experience any symptoms but did require medical intervention, and, therefore, this adverse event was given a "grade 2" score based on the common terminology criteria for adverse events [10].

In 2020, the U. S. Food and Drug Administration (FDA) approved nivolumab in combination with ipilimumab, as first-line treatment for adult patients with unresectable malignant pleural mesothelioma [11]. The approval of nivolumab and ipilimumab provided a chemotherapy-sparing option for the treatment of patients with previously untreatable, unresectable malignant pleural mesothelioma [11]. The dosage regimen approved for the treatment of malignant pleural mesothelioma was nivolumab 360 mg every three weeks and ipilimumab 1 mg/kg every six weeks.

A recent systematic review focused on chemotherapy-naive and immune checkpoint inhibitor-naive patients with malignancies revealed that the adverse event rate was high in patients on nivolumab-ipilimumab combination therapy. More than 80% of patients experienced any adverse event, nearly 30% of patients were forced to quit the regimen due to adverse events, and treatment-related death occurred in 0.7% of the cases [12]. Among the endocrine adverse events, hypothyroidism was the most frequently observed (13.1%), followed by hyperthyroidism (11.0%), hypopituitarism (9.5%), and adrenal insufficiency (4.8%) [12]. A retrospective cohort study on immune checkpoint inhibitor-induced thyroid dysfunction demonstrated that thyroid dysfunction occurred in nearly 40% of cases and that treatment with combination therapy with ipilimumab and nivolumab, or any CTLA-4 + PD-1/PD-L1 inhibitor combination, increased the likelihood of developing thyroid dysfunction, emphasizing the synergistic effect of combined PD-1 and CTLA-4 blockade in terms of toxicity [13]. Transient thyroiditis characterized by an initial thyrotoxic phase followed by a hypothyroid phase occurred in 8.3% of cases [13]. However, this study excluded patients with pre-existing thyroid dysfunction and those who did not have any documented thyroid function test at baseline.

In a study on the clinical characteristic of thyroid dysfunction induced by PD-I inhibitors (nivolumab or pembrolizumab), none of the patients who developed worsening of pre-existing hypothyroidism had an initial thyrotoxic phase. However, there was no mention of the underlying cause of the pre-existing hypothyroidism [14]. Patients who have undergone total thyroidectomy cannot have immune checkpoint-induced thyroiditis. However, a case of nivolumab-associated exacerbation of hypothyroidism, necessitating an increase in the dose of levothyroxine, has been reported in a patient who had previously undergone total thyroidectomy and radio-ablation. It was suggested that nivolumab negatively affected the absorption of levothyroxine in some way [15].

There have been previous reports of exacerbation of pre-existing autoimmune thyroid disease after therapy with nivolumab and ipilimumab. One report described a 68-year-old woman with subclinical hypothyroidism who developed full-blown hypothyroidism, requiring levothyroxine replacement after the first cycle of nivolumab. Her anti-thyroid peroxidase and anti-thyroglobulin antibodies were positive before and after nivolumab therapy [16]. Another report involved an 85-year-old man with Hashimoto’s disease who developed a thyroid storm after receiving ipilimumab and nivolumab and then became hypothyroid. There was no mention of the levothyroxine dose before and after the initiation of immune checkpoint inhibitor therapy [17]. In a previous case series, a 66-year-old woman with Hashimoto’s disease stable on low-dose levothyroxine (25 mcg daily) developed painless thyroiditis and required 50-75 mcg daily of levothyroxine after nivolumab therapy [18]. Another case described a 70-year-old man with subclinical hypothyroidism who developed full-blown hypothyroidism requiring levothyroxine replacement after receiving nivolumab [19].

Destructive thyroiditis seems to be a reliable indicator of tumor response to treatment following immune checkpoint inhibitor therapy. Data also suggest that pre-existing autoimmunity against the thyroid gland is critical for the development of thyroid IRAEs [20]. Recent studies involving thyroglobulin-immunized mice have implicated cytotoxic CD4+ T cells in the pathogenesis of immune checkpoint-induced destructive thyroiditis [20]. These studies are limited by the substantial manipulation required to induce autoimmunity in the mice models and mimic human pre-existing autoimmunity. Further studies are required [20].

## Conclusions

Immune checkpoint inhibitors can cause destructive thyroiditis followed by exacerbation of hypothyroidism in patients with pre-existing autoimmune hypothyroidism. This may be a CD4+ T cells-mediated cytotoxic event, but more studies are required to delineate this further. Patients with autoimmune thyroid disease require regular assessment of their levothyroxine replacement while on immune checkpoint inhibitor therapy. This case will add to the growing literature regarding thyroid IRAEs associated with the use of immune checkpoint inhibitors in patients with pre-existing autoimmune thyroid disease.
